# 38029 Helping Patients with Chronic Conditions Overcome the Challenges of High Deductible Health Plans

**DOI:** 10.1017/cts.2021.721

**Published:** 2021-03-30

**Authors:** Tiffany Y. Hu, Iman Ali, Jeffrey T. Kullgren

**Affiliations:** University of Michigan

## Abstract

ABSTRACT IMPACT: With a growing number of Americans enrolled in high-deductible health plans, patients, especially those with chronic conditions, face increasing cost-sharing burden. We aim to develop a novel behavioral intervention to help patients use consumer strategies to better manage their health care spending. OBJECTIVES/GOALS: To assess patient preferences to develop an intervention to encourage the use of cost-conscious strategies to manage out-of-pocket health care spending among high-deductible health plan (HDHP) enrollees with chronic conditions. METHODS/STUDY POPULATION: This mixed-methods study is first conducting semi-structured telephone interviews of up to 20 adults with one or more chronic conditions who are enrolled in an HDHP. Preliminary findings from these interviews are being used to inform the design of a national internet panel survey of at least 300 HDHP enrollees. Collectively, the interviews and survey will assess experiences of HDHP enrollees and their preferences for the content, design, format, and mode of an intervention to help them engage in cost-conscious health care behaviors. These findings will then be used to develop a novel behavioral intervention that will subsequently be pilot tested for acceptability, feasibility, and preliminary efficacy. RESULTS/ANTICIPATED RESULTS: Early interview data identified gaps in knowledge of health care consumer strategies among HDHP enrollees with low confidence in being able to engage in cost-conscious health care behaviors. Several participants indicated interest in an intervention to learn more about how to engage in cost-conscious strategies (e.g., putting aside money for anticipated health care expenses, comparing cost and quality for services at different places, and talking to providers about health care costs). Most early interview participants preferred an easily accessible technological intervention, such as a website or app. Interviews are continuing, and the national survey will be fielded in early 2021. DISCUSSION/SIGNIFICANCE OF FINDINGS: HDHP enrollees with chronic conditions could benefit from an intervention that helps them manage their high cost-sharing. Based on the results of interviews and a national survey, we will develop and pilot test a novel behavioral intervention to promote use of cost-conscious health care behaviors.

